# Latent classes based on clinical symptoms of military recruits with mental health issues and their distinctive clinical responses to treatment over a 6-month duration

**DOI:** 10.1192/j.eurpsy.2023.555

**Published:** 2023-07-19

**Authors:** D.-I. Jon, E.-H. Park, H. J. Hong, M. H. Jung, N. Hong

**Affiliations:** Psychiatry, Hallym University Sacred Heart Hospital, Anyang, Korea, Republic Of

## Abstract

**Introduction:**

In South Korea, all men at the age of 18 or older are required to serve at military for a certain period as an obligation. These recruits should be able to withstand psychological stress and pressures of rapid adaptation of the unique and new environment in military.

**Objectives:**

The current study attempted to identify distinct subgroups of patients referred for military service suitability and further to investigate whether there is a difference in clinical features such as treatment responsiveness and prognosis among those subgroups.

**Methods:**

Subjects were male patients aged 18 to 28 years who visited the department of psychiatry at the University Hospital for evaluating mental health problems related to military service. We conducted a latent profile analysis (LPA) using 10 clinical scales of MMPI-2 as an indicator variable to investigate the subgroups of subjects. The clinical state of subjects was assessed with CGI-S and GAF scale for three time point (0, 3, and 6 month).

**Results:**

The results showed that the best fitting model corresponded to a three-class model: each class was named ‘Class 1: mild maladjustment’, ‘Class 2: neurotic depression and anxiety’, and ‘Class 3: highly vulnerable and hypervigilant’ respectively. Subsequent analysis was also carried out to identify changes in clinical symptoms and functional level across treatment time of each subgroup identified in LPA. We demonstrated that the three subgroups displayed differential characteristics in treatment responsiveness and clinical course evaluated by CGI-S and GAF over a treatment period of 6 months. Three subgroups indicated significant differences in the number of medications prescribed as well. Class 3 had more antidepressants and anxiolytics on use than Class 1 and 2. Antipsychotic agents and a combination of three antidepressants were prescribed more frequently in Class 3 than in Class 1 and 2.

**Conclusions:**

While Class 1 and 2 significantly improved over 6 months, Class 3 showed little or no improvement in our clinical parameters even with more medications. This study has a clinical significance that it has classified qualitatively different subgroups within the sample by conducting LPA with clinical profiles of MMPI-2 and provides a basis for comprehensively understanding their differentiated clinical features. This study suggests clinical implications for treatment plan and intervention of each subgroup classified based on MMPI-2 clinical profiles of military recruits who might show maladjustment to serve.

**Disclosure of Interest:**

None Declared

